# A Novel Approach to Determining Bone Loss Through Serum Uric Acid Levels: A Retrospective Multicenter Cohort Analysis

**DOI:** 10.3390/jcm15083020

**Published:** 2026-04-15

**Authors:** Ahmet Aydin, Turkan Pasali Kilit, Seher Kir, Esref Arac, Osman Ozudogru, Nazmiye Serap Bicer, Gulbin Seyman Cetinkaya, Mehmet Selim Mamis, Kadem Arslan, Suleyman Bas, Hatice Beyazal Polat, Kamil Konur, Omer Faruk Alakus, Ihsan Solmaz, Gizem Zorlu Gorgulugil, Seyit Uyar, Sabin Goktas Aydin, Alihan Oral, Nurhayat Ozkan Sevencan, Ceren Cevik, Betul Danapinar, Cetin Uyanik, Osman Erinc, Ozgur Yilmaz, Sevtap Bakir Kaliber, Aynur Kamburoglu, Nizameddin Koca

**Affiliations:** 1 Department of Internal Medicine, Faculty of Medicine, Istanbul Medipol University, Istanbul 34214, Türkiye; cetinuyanik@gmail.com; 2 Department of Internal Medicine, Kutahya Health Sciences University, Kutahya 43020, Türkiye; turkan.pasalikilit@ksbu.edu.tr; 3 Department of Internal Medicine, Faculty of Medicine, Ondokuz Mayis University, Samsun 55270, Türkiye; seherkr@yahoo.com; 4 Department of Internal Medicine, Faculty of Medicine, Dicle University, Diyarbakir 21010, Türkiye; esrefarac@gmail.com; 5 Department of Internal Medicine, Faculty of Medicine, Erzincan Binali Yıldırım University, Erzincan 24100, Türkiye; osmanozudogru2@gmail.com; 6 Department of Internal Medicine, Kayseri City Hospital, Kayseri 38080, Türkiye; serapbicr@gmail.com; 7 Department of Internal Medicine, İzmir Atatürk Research and Training Hospital, İzmir 35360, Türkiye; gulbinseyman@gmail.com; 8 Department of Internal Medicine, Faculty of Medicine, Siirt University, Siirt 56100, Türkiye; dr.mehmetselim@outlook.com; 9 Department of Internal Medicine, Sancaktepe Sehit Prof. Dr. Ilhan Varank Training and Research Hospital, Istanbul 34785, Türkiye; kademarslan@hotmail.com (K.A.); suleymanbas.2012@gmail.com (S.B.); 10 Department of Internal Medicine, Faculty of Medicine, Recep Tayyip Erdogan University, Rize 53020, Türkiye; hatice.beyazalpolat@erdogan.edu.tr (H.B.P.); kamil.konur@erdogan.edu.tr (K.K.); 11 Department of Internal Medicine, Diyarbakır Gazi Yasargil Training and Research Hospital, Diyarbakır 21010, Türkiye; omerfaruk01@gmail.com (O.F.A.); ihsan2157@gmail.com (I.S.); 12 Department of Internal Medicine, Antalya Training and Research Hospital, Antalya 07100, Türkiye; gizemzorlug@gmail.com (G.Z.G.); seyituyar79@hotmail.com (S.U.); 13 Department of Medical Oncology, Kanuni Sultan Suleyman Training and Research Hospital, Istanbul 34303, Türkiye; drsabingoktas@gmail.com; 14 Department of Internal Medicine, Faculty of Medicine, Biruni University, Istanbul 34295, Türkiye; dr.alihanoral@gmail.com; 15 Department of Internal Medicine, Karabuk Training and Research Hospital, Karabuk 78200, Türkiye; dr_nurhayat@hotmail.com (N.O.S.); drcerencevik@gmail.com (C.C.); betul1danapinar@gmail.com (B.D.); 16 Department of Internal Medicine, Kanuni Sultan Suleyman Training and Research Hospital, Istanbul 34303, Türkiye; doctorerinc@gmail.com (O.E.); dr_ozguryilmaz@hotmail.com (O.Y.); 17 Department of Internal Medicine, Bursa City Hospital, Bursa 16250, Türkiye; drbakirsevtap@gmail.com (S.B.K.); aynururhann@gmail.com (A.K.); nkoca@yahoo.com (N.K.)

**Keywords:** bone mineral density, osteopenia, osteoporosis, serum uric acid

## Abstract

**Background:** Osteoporosis has a rising global incidence and social burden. Serum uric acid’s dual roles in oxidative stress and inflammation may influence bone health, but findings are inconsistent and require further research. This study aimed to evaluate the relationship between SUA levels and osteoporosis in a multicenter cohort obtained from different regions of Türkiye. **Methods:** This multi-center retrospective study included 3280 individuals, postmenopausal women and men aged 45 and older, from 16 centers in Türkiye. Individuals were excluded if they recently consumed alcohol, had severe renal dysfunction, certain hormonal or mineral disorders, specific medications, or certain menopausal statuses. Bone mineral density (BMD) at the hip and lumbar spine was measured using dual-energy X-ray absorptiometry (DXA), and participants were classified as normal or having osteopenia or osteoporosis based on T-score thresholds. **Results:** Overall, 34.8% were male, and 65.2% were female. For the lumbar spine, 36.8% had osteopenia, and 13.5% had osteoporosis; similarly, for the total hip, 40.8% had osteopenia, and 7.9% had osteoporosis. ROC analysis identified a threshold of 3.9 mg/dL serum uric acid (SUA) (AUC 0.374; *p* < 0.001), which was positively associated with both lumbar and total hip BMD. Osteoporosis rates were higher in patients with SUA < 3.9 mg/dL compared to those with SUA ≥ 3.9 mg/dL at the lumbar spine (29.1% vs. 14.2%, *p* < 0.001) and total hip sites (23.6% vs. 15.9%, *p* = 0.003). After adjustment for potential confounders, SUA was a significant independent predictor of osteoporosis in the lumbar spine (OR 0.70; *p* < 0.001) and the hip (OR 0.80; *p* < 0.001). **Conclusions:** Serum uric acid levels are inversely linked to bone mineral density and osteoporosis risk, indicating a potential role in bone health. However, due to study limitations, causal relationships remain unproven, and further research is needed.

## 1. Introduction

Osteoporosis is characterized by reduced bone mass, disrupted bone microarchitecture, and increased skeletal fragility, collectively resulting in diminished bone strength and an increased risk of fractures, with a 15–20% rise in mortality within one year of the fracture [1,2]. Osteoporosis remains clinically silent until a fracture occurs, with fractures reported in over 9 million of the 200 million newly diagnosed patients [1]. As life expectancy increases, such a fracture incidence causes a global health concern by imposing a major economic and social burden [3]. Decreased bone density is associated with age, gender, family history, and various factors such as smoking, malnutrition, low body mass index, low calcium intake, glucocorticoid usage, and alcohol consumption [4]. Although the exact mechanism remains unclear, oxidative stress alongside hormonal alterations and deficiencies in calcium and vitamin D is thought to suppress osteoblastic differentiation in bone cells while promoting osteoclastic differentiation, thereby enhancing bone resorption [5,6].

Serum uric acid (SUA), produced through xanthine oxidase during purine metabolism, can act as both an antioxidant and a pro-oxidant depending on the biological environment. While SUA significantly boosts circulating antioxidant defenses, elevated levels are associated with various health issues such as gout, kidney stones, hypertension, metabolic syndrome, cardiovascular diseases, and non-alcoholic fatty liver disease [7,8,9,10,11].

Growing evidence suggests SUA may have a broader role in oxidative stress and inflammation, sparking interest in its role in bone health and osteoporosis. While extracellular uric acid has antioxidant properties, its accumulation or cellular metabolism can increase oxidative stress and promote inflammation. This boosts osteoclast activity, inhibits osteoblasts, and, with uric acid-induced lower vitamin D synthesis and secondary hyperparathyroidism, can further enhance bone resorption [12].

The literature includes conflicting studies and meta-analyses. While some studies suggest that SUA is significantly positively correlated with bone mass [13,14,15], others do not support this hypothesis [16,17,18,19,20].

As the prevalence of osteoporosis continues to rise, there is an urgent need to identify reliable, low-cost biomarkers for early risk assessment and prevention. Serum uric acid is a promising candidate because of its key role in oxidative stress and inflammation, which influence bone remodeling. Nevertheless, studies on its effect on bone health are inconsistent, pointing to an important gap in our understanding.

We hypothesized that SUA levels are significantly associated with bone mineralization. To test this, we examined the relationship between serum uric acid and bone mineral density in postmenopausal women and men over 50 years across multiple regions of Türkiye to clarify its clinical relevance as a potential marker of osteoporosis risk.

## 2. Material Method

This multi-center retrospective study included 3280 individuals from 16 different centers in Turkey.

Individuals were not eligible for inclusion if they had consumed alcohol recently, defined as a daily intake of up to 14 g within the previous three months. Participants with marked renal dysfunction (eGFR < 30 mL/min/1.73 m^2^), known thyroid or parathyroid disease, or those receiving treatments that could influence hormonal balance or mineral metabolism, such as sex hormones, glucocorticoids, bisphosphonates, or calcium supplements, were also excluded. In addition, men younger than 50 years and women who had undergone surgical menopause or experienced menopause before age 40 were not included; however, a very small number of men aged <50 years (*n* = 4) were inadvertently enrolled, all of whom had normal BMD values. Patients with gout, a history of kidney stones, or those using medications likely to affect serum uric acid levels were excluded to avoid confounding of urate measurements. Patient demographics were obtained from hospital records. Laboratory findings were obtained at the time of the DXA scan.

Bone status at the total hip and lumbar spine (L1–L4) was evaluated by measuring bone mineral density (BMD, g/cm^2^) and assessing for osteoporosis using dual-energy X-ray absorptiometry (DXA) with a GE Lunar Prodigy Advance system, operated in accordance with the manufacturer’s guidelines. DXA measurements were obtained at the participating centers as part of routine clinical practice. BMD represents the quantity of mineralized bone within a defined skeletal area and was expressed both as an absolute value (g/cm^2^) and as a T-score. The T-score reflects the difference between an individual’s BMD and the mean peak bone mass of a healthy young reference population of the same sex, expressed in standard deviation units. Based on T-score values, participants were classified as having normal bone density (T ≥ −1.0), reduced bone mass (osteopenia; −2.5 < T < −1.0), or osteoporosis (T ≤ −2.5) [1].

This study was conducted in accordance with the ethical principles of the Declaration of Helsinki. Patients provided written informed consent, and the Local Ethics Committee approved the study in 20 February 2025 with decision number E-10840098-202.3.02-1588.

### Statistical Analysis

Statistical evaluation was carried out using SPSS software (version 24.0; IBM Corp., Chicago, IL, USA). The distribution of continuous variables was examined using both the Kolmogorov–Smirnov and Shapiro–Wilk tests. Descriptive data are presented as medians with interquartile ranges for variables showing non-normal distribution, while categorical variables are reported as counts and percentages. Group differences in categorical variables were assessed using the chi-square test. For comparisons involving continuous variables, the Mann–Whitney U test or Kruskal–Wallis test was applied, as appropriate. To determine factors independently associated with osteoporosis, binary logistic regression analysis was performed. Receiver operating characteristic (ROC) curve analysis was used to establish the most informative serum uric acid threshold for predicting osteoporosis, with diagnostic performance evaluated by calculating sensitivity, specificity, positive and negative predictive values, and the area under the ROC curve. Associations between independent variables and survival outcomes were expressed using 95% confidence intervals. All statistical tests were two-tailed, and a *p*-value below 0.05 was considered indicative of statistical significance.

## 3. Results

A total of 3280 patients were included; 1141 (34.8%) were male and 2139 (65.2%) were female. The median age was 62 years (range, 45–94). Diabetes mellitus was present in 1257 patients (38.3%), hypertension in 1510 (46.0%), dyslipidemia in 1505 (45.9%), and a history of stroke in 158 (4.8%). Coronary artery disease was observed in 405 patients (12.3%). Alcohol use and smoking were reported in 183 (5.6%) and 626 (19.1%) patients, respectively. Also, 23 patients (0.7%) had a BMI < 18.5 kg/m^2^, 436 (13.2%) had a BMI of 18.5–24.9 kg/m^2^, 1126 (34.3%) had a BMI of 25.0–29.9 kg/m^2^, and 1514 (46.1%) had a BMI ≥ 30.0 kg/m^2^.

Overall, 1625 patients (49.5%) had normal lumbar bone mineral density (T-score ≥ −1.0), 1207 (36.8%) had osteopenia (T-score between −1.0 and −2.5), and 444 (13.5%) had osteoporosis (T-score ≤ −2.5). Four patients with lumbar prostheses were excluded from the L1–L4 DXA T-score analysis.

A total of 1682 patients (51.3%) had normal total hip bone mineral density (T-score ≥ −1.0), 1337 (40.8%) had osteopenia (T-score between −1.0 and −2.5), and 258 (7.9%) had osteoporosis (T-score ≤ −2.5). Three patients with hip prostheses were excluded from the total hip DXA analysis (Table 1).

The median values of laboratory results as SUA, calcium, phosphate, albümin, ALP, PTH, and 25-OH-D were 5.1 mg/dL (range, 1.3–12.9), 9.6 mg/dL (7.09–12.5), 3.50 mg/dL (1.74–6.51), 4.39 g/dL (3.0–5.6), 78 U/L (10–384), 50.0 pg/mL (10–180), and 19.5 ng/mL (2.3–111.8), respectively (Supplementary Table S1).

**Figure 1:**
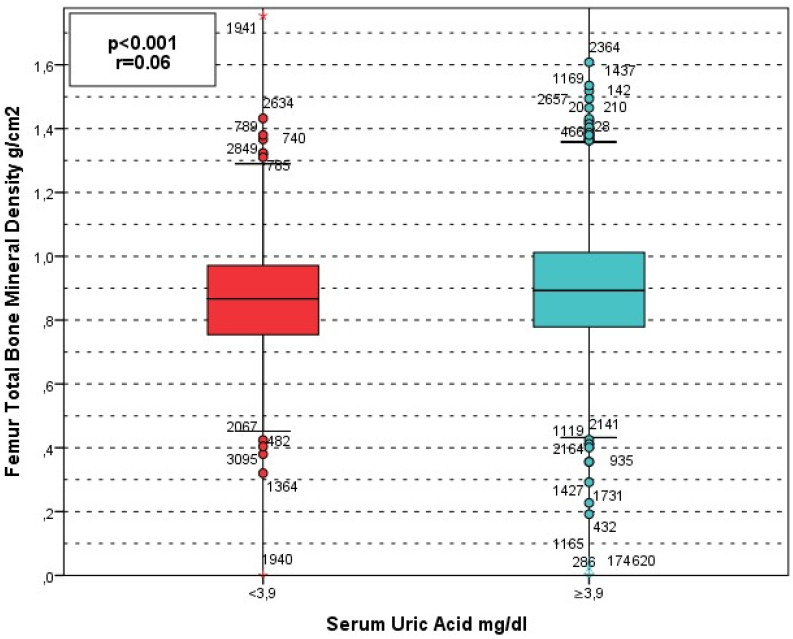
BMD femur and SUA. Circles (○) represent outliers, and asterisks (*) represent extreme outliers.

ROC curve analysis demonstrated an inverse discriminative ability of serum uric acid for the outcome, with an area under the curve (AUC) of 0.374 (standard error 0.014, 95% CI 0.346–0.402, *p* < 0.001). Using a cut-off value of 3.9 mg/dL, uric acid yielded a sensitivity of 74.1%, a specificity of 13.8%, and a corresponding Youden index of −0.1 (Figure 1).

For lumbar spine BMD, the proportion of patients with SUA < 3.9 mg/dL increased progressively across worsening BMD categories: 231 (14.2%) in the normal group, 224 (18.6%) in the osteopenia group, and 129 (29.1%) in the osteoporosis group (*p* < 0.001). Conversely, SUA ≥ 3.9 mg/dL was more frequent among individuals with normal BMD (1394 [85.8%]) and decreased among those with osteoporosis (315 [70.9%]) (*p* < 0.001).

**Figure 2:**
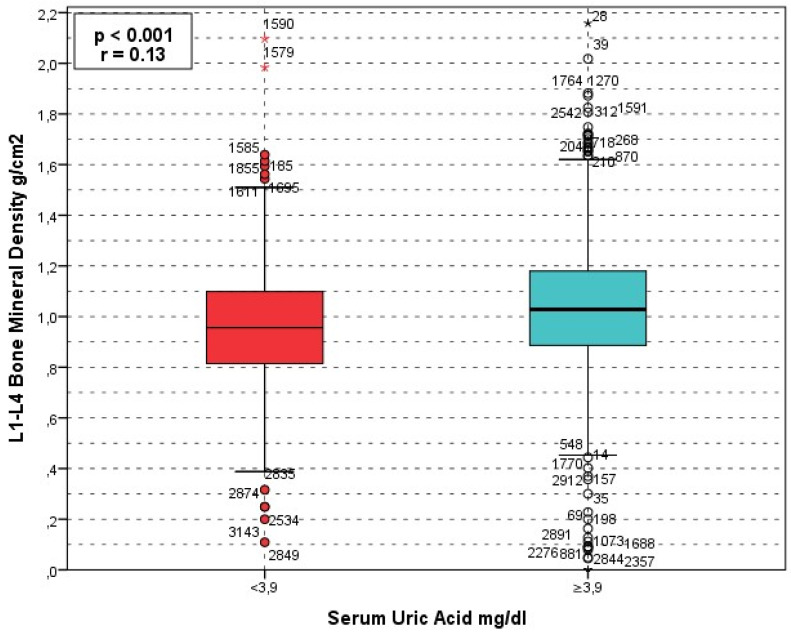
BMD lomber and SUA. Circles (○) represent outliers, and asterisks (*) represent extreme outliers.

**Figure 3:**
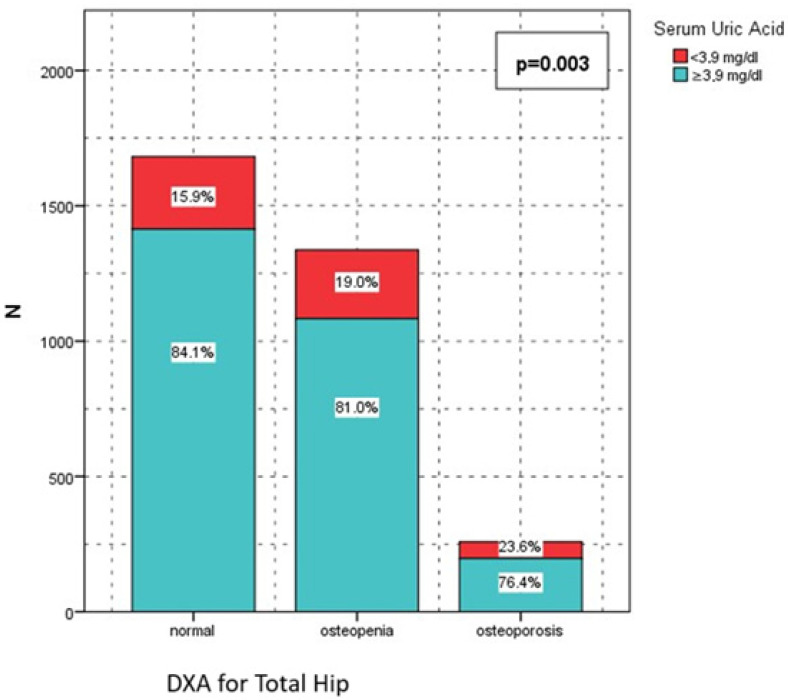
SUA and femur t score.

A similar pattern was observed for total hip BMD. The prevalence of SUA < 3.9 mg/dL was 268 (15.9%) in the normal group, 254 (19.0%) in the osteopenia group, and 61 (23.6%) in the osteoporosis group (*p* = 0.003). In contrast, SUA ≥ 3.9 mg/dL was most common among individuals with normal total hip BMD (1414 [84.1%]) and least common in those with osteoporosis (197 [76.4%]) (Figure 2 and Figure 3).

The inverse association between serum uric acid and osteoporosis prevalence remained consistent after stratification by sex (Supplementary Table S2).

BMI was significantly associated with both lumbar (L1–L4) and total hip DXA categories (both *p* < 0.001), with obese patients showing higher rates of normal BMD and lower rates of osteoporosis. Sex significantly influenced BMD distribution at both sites (*p* < 0.001), with females comprising a higher proportion of osteopenia and osteoporosis categories. Diabetes mellitus was significantly associated with BMD categories at the lumbar spine and total hip (*p* = 0.005 and *p* = 0.001, respectively), with higher osteoporosis rates observed in patients with diabetes. Hypertension was significantly associated with lumbar BMD (*p* = 0.017) but not with total hip BMD (*p* = 0.57). Active alcohol use and active smoking were significantly associated with BMD categories at both anatomical sites (all *p* ≤ 0.03), with osteoporosis occurring more frequently among alcohol users and smokers. Serum uric acid < 3.9 mg/dL accounted for a disproportionately higher share of osteoporosis cases, particularly at the lumbar spine (29.1% vs. 14.2% in the normal BMD group; *p* < 0.001) and total hip (23.6% vs. 15.9%; *p* = 0.003). In contrast, 25-hydroxyvitamin D levels were not significantly associated with BMD categories at either site (*p* = 0.19 and *p* = 0.93) (Table 2).

**Figure 4:**
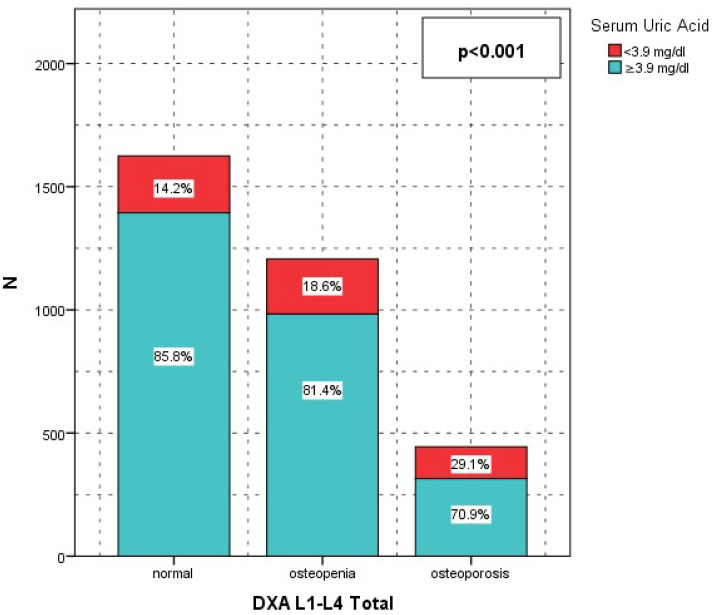
SUA lomber t score.

The relationship between BMD (g/cm^2^) and laboratory markers was assessed using the Kruskal–Wallis test which revealed that there was a significant correlation between Lumbar BMD (g/cm^2^) and age (*p* < 0.001), SUA (*p* < 0.001), ALP (*p* < 0.001), serum Calcium (*p* = 0.02), albümin (*p* < 0.001), and PTH (*p* < 0.001) (Figure 4). While no signiificant relation was observed between lumbar BMD and phosphore (*p* = 0.3), and vitamin D (*p* = 0.5). The analysis for total hip BMD revealed that, age (*p* < 0.001), SUA (*p* < 0.001), calcium (*p* < 0.001), albümin (*p* < 0.001), Parathormon (*p* < 0.001), phosphore (*p* = 0.039), and ALP (*p* = 0.001) significantly linked with total hip BMD. Meanwhile vitamin D was not significantly linked with total hip BMD (*p* = 0.9).

Lumbar spine BMD (g/cm^2^) increased progressively across serum uric acid quartiles, with median values of 0.958 (IQR 0.289), 0.985 (0.305), 1.034 (0.281), and 1.069 (0.279) from the lowest to highest quartile, respectively (*p* < 0.001). A similar trend was observed for total hip BMD, with median values of 0.864 (0.223), 0.876 (0.222), 0.902 (0.237), and 0.915 (0.253), respectively (*p* < 0.001). Consistently, mean rank analysis demonstrated a stepwise increase in BMD across quartiles for both lumbar spine (χ^2^ = 86.9, *p* < 0.001) and total hip (χ^2^ = 32.4, *p* < 0.001).

In multivariable logistic regression analysis, age was an independent risk factor for both lumbar and total hip osteoporosis (lumbar: OR 1.047, 95% CI 1.033–1.062; total hip: OR 1.098, 95% CI 1.079–1.117; both *p* < 0.001). Serum uric acid was independently and inversely associated with osteoporosis at both sites (lumbar: OR 0.700, 95% CI 0.635–0.772; total hip: OR 0.807, 95% CI 0.722–0.901; *p* < 0.001). Parathyroid hormone was an independent predictor of increased osteoporosis risk at both the lumbar spine and total hip (OR 1.010 for both; *p* < 0.001). Alkaline phosphatase was significantly associated only with lumbar osteoporosis (OR 1.005, 95% CI 1.001–1.009; *p* = 0.019). In contrast, albumin, BMI, calcium, and phosphorus were not independent determinants of osteoporosis at either anatomical site (all *p* > 0.05) (Table 3).

## 4. Discussion

Osteoporosis is a common global public health issue, with its prevalence increasing significantly with age and sex as menopausal status is an important determinant of both serum uric acid metabolism and bone mineral density. Estrogen decline after menopause is associated with increased SUA levels and accelerated bone loss, suggesting a shared biological pathway [21].

A report using data from the U.S. National Health and Nutrition Examination Survey found that, in 2017–2018, 12.6% of individuals had osteoporosis at the femoral neck, lumbar spine, or both, with women affected at a rate of 19.6% and men at a rate of 4.4% [19]. Reports from different areas have reported that the prevalence of osteoporosis of the femoral neck in men ranges from 4.4% to 11.7%, whereas the corresponding rates in women range from 19.6% to 23.1% [21,22]. In Turkey, the FRACTURK study initially included 26,424 individuals aged ≥50 years who were screened for a history of fracture, and, among a subcohort of 1971 participants who underwent bone mineral density measurements, osteopenia was identified in 49.6% and osteoporosis in 25.6% [23].

In our cohort, lumbar spine osteoporosis was present in 444 patients (13.5%) overall, including 85/1141 (7.4%) and 359/2139 women (16.7%), while total hip osteoporosis was observed in 258 patients (7.9%) overall, including 80/1141 men (7.0%) and 178/2139 women (8.3%). Although most epidemiological studies, including NHANES, report site-specific prevalence based on femoral neck measurements, our analysis was based on total hip BMD.

Various factors, including metabolic- and lifestyle-related comorbidities, have been identified as risk factors for developing osteoporosis. Worldwide, a high prevalence of OP was found in patients with T2DM [24,25]. However, regarding diabetes mellitus Zeng et al. reported no significant difference between the osteoporosis (22.2%) and non-osteoporosis (28.1%) groups (*p* = 0.274) [26].

Hypertension and osteoporosis share a connected biological pathway [27]. The NHANES analysis reported a progressive increase in hypertension prevalence with worsening bone density [28]. Zeng et al. showed higher systolic blood pressure in osteoporosis (*p* < 0.001) rather than diagnosis-based comparisons, and Wang et al. found comparable hypertension rates across groups (*p* = 0.799) [26,29].

Smoking and alcohol consumption demonstrated the most consistent pattern, with both Zeng et al. and Wang et al. reporting significantly lower smoking and alcohol use in osteoporosis groups (smoking 38.8% vs. 43.5%, *p* = 0.026; alcohol 40.3% vs. 61.8%, *p* < 0.001; both *p* < 0.001 in Wang et al.), indicating a shared trend toward reduced exposure to these lifestyle factors in individuals with lower bone mineral density [26,29].

In our study, the prevalence of diabetes mellitus significantly declined as bone mineral density worsened, dropping from 40.7% to 37.4% in lumbar DXA and from 40.4% to 32.2% in total hip DXA (*p* = 0.005 and *p* = 0.001, respectively). Hypertension showed a slight decrease across lumbar categories, from 59.4% to 54.4% (*p* = 0.017), but remained relatively unchanged across total hip categories, from 55.3% to 58.4% (*p* = 0.57). Active smoking decreased notably with worsening BMD at both sites, from 29.6% to 14.8% (*p* < 0.001 for both), and active alcohol use also markedly declined, from 8.4% to 4.8% in lumbar and from 9.7% to 3.7% in total hip DXA categories (*p* = 0.03 and *p* < 0.001, respectively).

Qu et al. found that higher serum PTH levels were causally and inversely associated with BMD across multiple skeletal sites, supporting a site- and age-specific role of PTH in the development of osteoporosis [30]. Salamat et al. reported no significant overall relationship between routine biochemical parameters (calcium, phosphorus, ALP, vitamin D, magnesium) and BMD, although limited site-specific correlations were observed [31]. Yilmaz et al. showed that bone resorption markers had stronger diagnostic performance for osteoporosis than bone formation markers, while ALP-related markers were relatively weak discriminators [32].

Our study showed that lumbar osteoporosis significantly related to age, ALP, serum calcium, albumin, and parathyroid hormone (all *p* ≤ 0.02). Phosphorus and vitamin D showed no significant link. For total hip osteoporosis, significant links were with age, SUA, calcium, albumin, parathormone, phosphorus, and ALP (all *p* ≤ 0.039), while vitamin D was not. Multivariable regression found older age and higher parathyroid hormone as independent risk factors at both sites. ALP was an independent predictor only for lumbar osteoporosis; albumin, calcium, phosphorus, and BMI were not independently linked.

Uric acid, a by-product of purine metabolism, has gained increasing attention due to its antioxidant properties and potential protective role in bone metabolism, as it scavenges reactive oxygen species (ROS) and inhibits NADPH oxidase activity, thereby protecting osteoblasts from oxidative damage, as demonstrated in experimental and clinical studies [16,18,33]. A large meta-analysis conducted by Shen et al. showed a significant positive correlation between SUA and BMD, the risk of osteoporosis was significantly lower in patients with higher SUA levels (OR  =  0.59; 95% CI: 0.52, 0.67; *p*  <  0.001; I2  =  30.3%) [33]. Similarly, Xu et al. showed a significant association between BMD and SUA by adjusting for BMI, gender, age, vitamin D, and blood urea nitrogen. Their analysis indicated that a 0.0286 g/cm^2^ increase in BMD was observed per 100 μmol/L rise in SUA levels [34]. In another study, after adjustment for all covariates, higher serum uric acid levels were independently associated with a reduced risk of osteoporosis or osteopenia, with each unit increase in SUA associated with a 0.3% lower odds, and individuals in the highest quartile demonstrating a 34.9% lower risk compared with the lowest quartile, while restricted cubic spline analysis confirmed a significant linear inverse dose–response relationship without evidence of non-linearity [35]. A large cross-sectional study including American and Chinese adults demonstrated similar results. Additionally, their nonlinear analysis identified saturation thresholds, with maximal protective effects observed at approximately 410–452 μmol/L in Americans and 429–468 μmol/L in Chinese individuals [36]. Liu et al. reported that individuals with the highest serum uric acid levels had a 49% lower risk of osteoporosis compared with those in the lowest group (OR = 0.513). The reduction in osteoporosis risk became evident at approximately 5.3 mg/dL. Furthermore, Mendelian randomization analysis confirmed a causal protective effect of higher serum uric acid levels on osteoporosis risk (OR = 0.870) [37].

In contrast to the predominantly protective associations reported in prior literature, Li et al. found no significant association between serum uric acid levels and lumbar spine bone mineral density in a cross-sectional study of 6704 adult men after adjustment for confounders (β = −0.003, 95% CI −0.007 to 0.002). However, the inclusion of a relatively young population, with over half of participants under 40 years of age, and the exclusive assessment of lumbar spine BMD may have limited the ability to detect associations at higher-risk ages or other clinically relevant skeletal sites such as the total hip, potentially explaining the null findings [18]. Also, Kang et al. demonstrated no independent relationship between serum uric acid (SUA) levels and bone mineral density at either the lumbar spine or hip. Although higher SUA tertiles were associated with numerically higher BMD values, these differences were not statistically significant, and multivariable regression analyses, adjusting for age, BMI, body composition, and metabolic parameters, confirmed the absence of an independent association [38].

In our study, serum uric acid (SUA) showed a significant positive association with bone mineral density and an inverse association with osteoporosis risk. ROC analysis identified a SUA threshold of 3.9 mg/dL (AUC 0.374, 95% CI 0.346–0.402, *p* < 0.001), and patients with SUA ≥ 3.9 mg/dL had significantly higher lumbar and total hip BMD values together with lower osteoporosis prevalence compared with those below this level (lumbar *p* < 0.001; total hip *p* = 0.003). The relatively low AUC indicates limited discriminative performance of serum uric acid. However, complementary analyses using continuous BMD values demonstrated a significant and consistent increase in both lumbar spine and total hip BMD across SUA quartiles, supporting a biologically relevant dose–response relationship rather than a threshold-based effect. In the correlation analyses, SUA was significantly associated with both lumbar and total hip BMD (*p* < 0.001 for both). After adjustment for potential confounders, including age, BMI, albumin, calcium, phosphorus, ALP, and parathyroid hormone, serum uric acid remained independently associated with osteoporosis risk at both skeletal sites. each 1 mg/dL increase in SUA was associated with a 30% reduction in the odds of lumbar osteoporosis (OR 0.700, 95% CI 0.635–0.772, *p* < 0.001) and a 19% reduction in the odds of total hip osteoporosis (OR 0.807, 95% CI 0.722–0.901, *p* < 0.001).

Given the retrospective observational design, the findings demonstrate association rather than causation, and causal relationships cannot be inferred. This study has several limitations that should be acknowledged. First, serum uric acid levels were measured at a single time point, and potential biological fluctuations over time could not be assessed. Because uric acid levels may vary with hydration status, metabolic stress, and short-term dietary influences, a single measurement may not fully reflect long-term exposure. In addition, dietary factors that could act as potential confounders were not comprehensively evaluated. The consumption of purine-rich foods, such as shellfish and organ meats, was not quantified, which may have influenced serum uric acid levels. In addition, dietary and lifestyle-related factors that may influence both serum uric acid levels and bone metabolism were not comprehensively evaluated. Variables such as dietary habits, physical activity, and supplementation (including calcium and vitamin D) were not available in the dataset. Although we performed multivariable analyses adjusting for key clinical and biochemical parameters, residual confounding due to unmeasured lifestyle factors cannot be fully excluded and should be considered when interpreting the observed associations. Another limitation is the multicenter acquisition of DXA measurements. Inter-scanner variability and potential differences in calibration cannot be fully excluded. However, such variability would be expected to introduce non-differential measurement error and bias the results toward the null, rather than account for the observed associations.

Despite these limitations, the current study has many strengths that impact current knowledge. One of the major strengths of our study is its multicenter design, which incorporates data from different geographic regions of Türkiye and includes both men and postmenopausal women, thereby enhancing national representativeness and generalizability. In addition, considering the multifactorial nature of osteoporosis, we performed comprehensive adjustments for key determinants of bone metabolism, including age, body mass index, albumin, calcium, phosphorus, alkaline phosphatase, and parathyroid hormone. Furthermore, the identification of a clinically relevant threshold through ROC analysis may influence future bone health assessment algorithms. Moreover, in quartile-based analysis, higher SUA levels were associated with progressively higher BMD values at both skeletal sites, supporting a biologically relevant dose–response relationship beyond categorical thresholds.

## 5. Conclusions

Collectively, these findings position our study beyond a purely correlational analysis and provide clinically interpretable evidence regarding the potential relationship between serum uric acid and bone health.

## Figures and Tables

**Table 1:** Patient Characteristics.

Patient Characteristics	*N* = 3280	%
Age median (range)	62 (45–94)	
Gender		
Male	1141	34.8
Female	2139	65.2
Diabetes mellitus		
Yes	1257	38.3
No	2022	61.6
Missing data	1	0.01
Hypertension		
Yes	1510	46.0
No	1160	35.4
Missing data	609	18.6
Dyslipidemia		
Yes	1505	45.9
No	1773	54.1
History of fragility fracture		
Yes	325	9.9
No	2344	71.5
Missing data	611	18.6
History of stroke		
Yes	158	4.8
No	2394	73
Missing data	611	18.6
Coronary artery disease		
Yes	405	12.3
No	2265	69.1
Missing Data	609	18.6
Alcohol use		
Yes	183	5.6
No	2483	75.7
Missing data	613	18.7
Smoking		
Yes	626	19.1
No	2041	62.2
Missing data	612	18.2
Serum uric acid		
<3.9	584	17.8
≥3.9	2696	82.2
BMI category		
<18.5 kg/m^2^	23	0.7
18.5–24.9 kg/m^2^	436	13.2
25.0–29.9 kg/m^2^	1126	34.3
≥30.0 kg/m^2^	1514	46.1
Not assessed	181	5.7
T-scores for DXA L1–L4		
≥−1.0	1625	49.5
−1.0–(−2.5)	1207	36.8
≤−2.5	444	13.5
Not assessed	4	0.01
T-scores for DXA total hip		
≥−1.0	1682	51.3
−1.0–(−2.5)	1337	40.8
≤−2.5	258	7.9
Not assessed	3	0.01

**Table 2:** Demographic, clinical, and biochemical characteristics according to bone mineral density categories based on DXA T-scores at the lumbar spine and total hip.

Category	T-Scores for L1-L4			*p*	T Scores for Total Hip			*p*
	≥−1.0	−1.0–(−2.5)	≤−2.5		≥−1.0	−1.0–(−2.5)	≤−2.5	
	*N* (%)	*N* (%)	*N* (%)		*N* (%)	*N* (%)	*N* (%)	
BMI				<0.001				<0.001
<18.5	5 (0.3)	11 (1.0)	7 (1.7)		2 (0.1)	10 (0.8)	11 (4.4)	
18.5–24.9	183 (11.7)	163 (14.5)	89 (21.9)		142 (9)	216 (17.1)	77 (31)	
24.9-29.9	539 (34.5)	436 (38.8)	150 (36.9)		549 (34.6)	486 (38.5)	91 (36.7)	
>30	836 (53.5)	515 (45.8)	161 (39.6)		892 (56.3)	551 (43.6)	69 (27.8)	
Gender				<0.001				<0.001
Male	718 (44.2)	338 (28.0)	85 (19.1)		656 (39)	405 (30.3)	80 (31)	
Female	907 (55.8)	869 (72.0)	359 (80.9)		1026 (61)	932 (69.7)	178 (69)	
Diabetes mellitus				0.005				0.001
Yes	662 (40.7)	427 (35.4)	166 (37.4)		679 (40.4)	494 (36.9)	83 (32.2)	
No	963 (59.3)	780 (64.6)	277 (62.4)		1003 (59.6)	843 (63.1)	174 (67.4)	
Hypertension				0.017				0.57
Yes								
No	697 (59.4)	582 (54.3)	229 (54.4)		687 (55.3)	680 (57.5)	142 (58.4)	
Missing data	477 (40.6)	490 (45.7)	191 (45.4)		556 (44.7)	502 (42.4)	101 (41.6)	
Active Alcohol use				0.03				<0.001
Yes	99 (8.4)	64 (6.0)	20 (4.8)		121 (9.7)	53 (4.5)	9 (3.7)	
No	1075 (91.6)	1007 (93.9)	397 (95.2)		1120 (90.2)	1128 (95.5)	233 (96.3)	
Active Smoking				<0.001				<0.001
Yes								
No	368 (29.6)	220 (18.6)	36 (14.8)		368 (29.6)	220 (18.6)	36 (14.8)	
Missing data	875 (70.4)	959 (81.3)	207 (85.2)		875 (70.4)	959 (81.4)	207 (85.2)	
Serum uric acid				<0.001				0.003
<3.9	231 (14.2)	224 (18.6)	129 (29.1)		268 (15.9)	254 (19)	61 (23.6)	
≥3.9	1394 (85.8)	983 (81.4)	315 (70.9)		1414 (84.1)	1083 (81)	197 (76.4)	
25-OH D,				0.19				0.93
<12 ng/mL	383 (23.7)	288 (24.1)	123 (28.3)		403 (24)	326 (24.7)	65 (25.7)	
12–20 ng/mL	444 (27.4)	346 (28.9)	106 (24.4)		472 (28.1)	355 (26.9)	68 (26.9)	
>20 ng/mL	792 (48.9)	563 (47.0)	206 (47.4)		803 (47.9)	640 (48.4)	120 (47.4)	

**Table 3:** Multivariable logistic regression analysis evaluating clinical and biochemical factors associated with osteoporosis defined by DXA T-scores at the lumbar spine and total femur.

	Factors	Coefficient (β)	Wald χ^2^	*p* Value	OR	95% CI
**Lumbar total osteoporosis**	Age (years)	0.046	45.69	<0.001	1.047	1.033–1.062
	Uric acid (mg/dL)	−0.356	50.68	<0.001	0.700	0.635–0.772
	Parathyroid hormone (pg/mL)	0.010	19.40	<0.001	1.010	1.005–1.014
	Albumin (g/dL)	−0.186	1.67	0.197	0.830	0.626–1.101
	Body mass index (kg/m^2^)	0.000	2.54	0.111	1.000	1.000–1.000
	Alkaline phosphatase (U/L)	0.005	5.50	0.019	1.005	1.001–1.009
	Calcium (mg/dL)	0.056	0.20	0.653	1.057	0.830–1.347
	Phosphorus (mg/dL)	0.150	1.90	0.168	1.162	0.939–1.439
**Hip total osteoporosis**	Age (years)	0.093	113.71	<0.001	1.098	1.079–1.117
	Uric acid (mg/dL)	−0.215	14.54	<0.001	0.807	0.722–0.901
	Parathyroid hormone (pg/mL)	0.010	15.80	<0.001	1.010	1.005–1.015
	Albumin (g/dL)	−0.309	3.03	0.082	0.734	0.518–1.040
	Body mass index (kg/m^2^)	0.000	0.81	0.367	1.000	1.000–1.000
	Alkaline phosphatase (U/L)	0.003	1.36	0.243	1.003	0.998–1.008
	Calcium (mg/dL)	−0.218	2.40	0.121	0.804	0.611–1.059
	Phosphorus (mg/dL)	−0.025	0.04	0.850	0.976	0.755–1.260

## Data Availability

The raw data supporting the conclusions of this article will be made available by the authors on request.

## Supplementary Materials

The following supporting information can be downloaded at: https://www.mdpi.com/article/10.3390/jcm15083020/s1, Figure S1: Study population by province; Table S1: Baseline laboratory characteristics of the overall study cohort; Table S2: Sex-stratified clinical characteristics and bone mineral density distribution according to serum uric acid categories.

## References

[1] Kanis J.A. (2007). Assessment of Osteoporosis at the Primary Health-Care Level.

[2] Johnston C.B., Dagar M. (2020). Osteoporosis in older adults. Med. Clin. North. Am..

[3] Compston J.E., McClung M.R., Leslie W.D. (2019). Osteoporosis. Lancet.

[4] Sànchez-Riera L., Carnahan E., Vos T., Veerman L., Norman R., Lim S.S., Hoy D., Smith E., Wilson N., Nolla J.M. (2014). The global burden attributable to low bone mineral density. Ann. Rheum. Dis..

[5] Kimball J.S., Johnson J.P., Carlson D.A. (2021). Oxidative stress and osteoporosis. J. Bone Jt. Surg. Am..

[6] Bai X.C., Lu D., Bai J., Zheng H., Ke Z.Y., Li X.M., Luo S.Q. (2004). Oxidative stress inhibits osteoblastic differentiation of bone cells by ERK and NF-κB. Biochem. Biophys. Res. Commun..

[7] Dalbeth N., Gosling A.L., Gaffo A., Abhishek A. (2021). Gout. Lancet.

[8] Ames B.N., Cathcart R., Schwiers E., Hochstein P. (1981). Uric acid provides an antioxidant defense in humans against oxidant- and radical-caused aging and cancer: A hypothesis. Proc. Natl. Acad. Sci. USA.

[9] Feig D.I., Kang D.H., Johnson R.J. (2008). Uric acid and cardiovascular risk. N. Engl. J. Med..

[10] Kanbay M., Jensen T., Solak Y., Le M., Roncal-Jimenez C., Rivard C., Lanaspa M.A., Nakagawa T., Johnson R.J. (2016). Uric acid in metabolic syndrome: From an innocent bystander to a central player. Eur. J. Intern. Med..

[11] Aydin A., Goktas Aydin S. (2024). Associations of serum uric acid levels and anthropometric parameters with non-alcoholic fatty liver disease in healthy individuals: Innovative insights from a cross-sectional study. Curr. Med. Res. Opin..

[12] Lin K.M., Lu C.L., Hung K.C., Wu P.C., Pan C.F., Wu C.J., Syu R.S., Chen J.S., Hsiao P.J., Lu K.C. (2019). The paradoxical role of uric acid in osteoporosis. Nutrients.

[13] Chen F., Wang Y., Guo Y., Wang J., Yang A., Lv Q., Liu Y., Ma G., Liu Y., Wang D. (2019). Specific higher levels of serum uric acid might have a protective effect on bone mineral density within a Chinese population over 60 years old: A cross-sectional study from northeast China. Clin. Interv. Aging.

[14] Lee J.W., Kwon B.C., Choi H.G. (2021). Analyses of the relationship between hyperuricemia and osteoporosis. Sci. Rep..

[15] Li J.Y., Lee J.I., Lu C.C., Su Y.D., Chiu C.T., Chen S.C., Geng J.H., Chen C.H. (2022). Hyperuricemia and its association with osteoporosis in a large Asian cohort. Nutrients.

[16] Dalbeth N., Horne A., Mihov B., Gamble G.D., Merriman T.R., Stamp L.K., Reid I.R. (2021). Elevated urate levels do not alter bone turnover markers: Randomized controlled trial of inosine supplementation in postmenopausal women. Arthritis Rheumatol..

[17] Hu Z., Zhang L., Lin Z., Liu W., Zhang J., Li W., Chu Y. (2021). Prevalence and risk factors for bone loss in rheumatoid arthritis patients from South China: Modeled by three methods. BMC Musculoskelet. Disord..

[18] Li X., Li L., Yang L., Yang J., Lu H. (2021). No association between serum uric acid and lumbar spine bone mineral density in US adult males: A cross-sectional study. Sci. Rep..

[19] Sarafrazi N., Wambogo E.A., Shepherd J.A. (2021). Osteoporosis or low bone mass in older adults: United States, 2017–2018. NCHS Data Brief.

[20] Salari N., Ghasemi H., Mohammadi L., Rabieenia E., Shohaimi S., Mohammadi M. (2021). The global prevalence of osteoporosis in the world: A comprehensive systematic review and meta-analysis. J. Orthop. Surg. Res..

[21] Vorobeľová L., Danková Z., Candráková-Čerňanová V., Falbová D., Cvíčelová M., Beňuš R., Siváková D. (2019). Association of the ESR1 polymorphism with menopause and MLXIPL genetic variant influence serum uric acid levels in Slovak midlife women. Menopause.

[22] Borgström F., Karlsson L., Ortsäter G., Norton N., Halbout P., Cooper C., Lorentzon M., McCloskey E.V., Harvey N.C., Javaid M.K. (2020). Fragility fractures in Europe: Burden, management and opportunities. Arch. Osteoporos..

[23] Tuzun S., Eskiyurt N., Akarirmak U., Saridogan M., Senocak M., Johansson H., Kanis J.A. (2012). Incidence of hip fracture and prevalence of osteoporosis in Turkey: The FRACTURK study. Osteoporos. Int..

[24] Liu X., Chen F., Liu L., Zhang Q. (2023). Prevalence of osteoporosis in patients with diabetes mellitus: A systematic review and meta-analysis of observational studies. BMC Endocr. Disord..

[25] Kalra S., Joshi A., Kapoor N. (2022). Osteoporosis and diabetes: The dual pandemics. J. Pak. Med. Assoc..

[26] Zeng J., Li T., Pan Z., Liu Q., He J., Cai X., Gong M., Deng X., Gong Y., Li N. (2025). Role of TyG, TyG-BMI and METS-IR in osteoporosis risk among older men: A retrospective cohort study. Asia Pac. J. Clin. Nutr..

[27] Gutierrez Y., Shaju R.A., Sepulveda A., Coban S. (2025). Exploring the interplay between hypertension and osteoporosis: A narrative review. Cureus.

[28] Shi L., Yu X., Pang Q., Chen X., Wang C. (2022). The associations between bone mineral density and long-term risks of cardiovascular disease, cancer, and all-cause mortality. Front. Endocrinol..

[29] Wang H., Wang Q., He B., Bai J., Wang N., Liu D., Cai J., Wang J., Xie Q. (2026). Osteoporosis and cardiac remodeling in middle-aged and older adults: A cross-sectional study. Sci. Rep..

[30] Qu Z., Yang F., Hong J., Wang W., Yan S. (2020). Parathyroid hormone and bone mineral density: A Mendelian randomization study. J. Clin. Endocrinol. Metab..

[31] Salamat M.R., Momeni S., Rastegari A.A. (2023). Relation between biochemical parameters and bone density in postmenopausal women with osteoporosis. Adv. Biomed. Res..

[32] Yilmaz N., Bayram M., Erbağci A.B., Kilinçer M.S. (1999). Diagnostic value of biochemical markers of bone turnover and postmenopausal osteoporosis. Clin. Chem. Lab. Med..

[33] Shen L., Meng F., Jiang Q., Sheng J., Feng H., Wang Y., Long H., Xie D., Yang T., Ding X. (2025). Association of serum uric acid level with bone mineral density and the risk of osteoporosis: A dose-response meta-analysis. Int. J. Rheum. Dis..

[34] Xu M.Z., Lu K., Yang X.F., Ye Y.W., Xu S.M., Shi Q., Gong Y.Q., Li C. (2023). Association between serum uric acid levels and bone mineral density in patients with osteoporosis: A cross-sectional study. BMC Musculoskelet. Disord..

[35] Xiu Z., Gao Z., Luo L. (2025). The triangular relationship of serum uric acid, osteoporosis or osteopenia, and body mass index for men and postmenopausal women. Sci. Rep..

[36] Li X., Peng Y., Chen K., Zhou Y., Luo W. (2025). Association between serum uric acid levels and bone mineral density in Chinese and American populations: A cross-sectional study. Sci. Rep..

[37] Liu Q.P., Zhang L.X., Luo J.H., Gao H.Z., Wang K. (2026). Association between serum uric acid levels and osteoporosis risk: Evidence from a cross-sectional study and Mendelian randomization analysis. Clin. Rheumatol..

[38] Kang S., Kwon D., Lee J., Chung Y.J., Kim M.R., Namkung J., Jeung I.C. (2021). Association between serum uric acid levels and bone mineral density in postmenopausal women: A cross-sectional and longitudinal study. Healthcare.

