# Supplementary material for: A Novel Approach to Determining Bone Loss Through Serum Uric Acid Levels: A Retrospective Multicenter Cohort Analysis
Source: J Clin Med. 2026 Apr 15;15(8):3020. doi: 10.3390/jcm15083020 (PMC13116610; doi:10.3390/jcm15083020)
Supplement: Supplementary file 1 [file jcm-15-03020-s001.zip › Suppl. Table S1. Laboratory findings.pdf]

Supplementary Table S1: Baseline laboratory characteristics of the overall study cohort

**Laboratory Findings**	Median (range)
Uric acid mg/dL **(**median (range)	5,1(1,3-12,9)
Aspartate aminotransferase, U/L **(**median (range)	19 (4-292)
Alanine aminotransferase, U/L **(**median (range)	18(1-395)
Alkaline phosphatase, U/L **(**median (range)	78 (10-384)
Serum albumin, g/dL **(**median (range)	4,39(3-5,6)
Calcium, mg/dL (median (range)	9,6(7,09-12,5)
Parathyroid hormone, pg/mL(median (range)	50,0 (10-180)
25-hydroxyvitamin D, ng/mL (median (range)	19,5 (2,3-111,83)
Hemoglobin, g/dL (median (range)	13,6(8-19)
Platelet count, $\times 10^9$/L (median (range)	256(103-663)
Phosphate, mg/dL (median (range)	3,50 (1,74-6,51)
Creatinine mg/dl	0,80 (0,34-1,60)
