# Supplementary material for: A Novel Approach to Determining Bone Loss Through Serum Uric Acid Levels: A Retrospective Multicenter Cohort Analysis
Source: J Clin Med. 2026 Apr 15;15(8):3020. doi: 10.3390/jcm15083020 (PMC13116610; doi:10.3390/jcm15083020)
Supplement: Supplementary file 1 [file jcm-15-03020-s001.zip › Suppl. Table S2.pdf]

Supplementary Table S2: Sex-stratified clinical characteristics and bone mineral density distribution according to serum uric acid categories

	**Male (n=1,141)**			**Female (n=2,139)**		
**Age, years**	62 (45–92)*			62 (45–71)		
**SUA mg/dL**	5.4 (1.3–11.3)			4.9 (1.5–12.9)		
**Lumbar spine BMD (g/cm²)**	1.090 (0.49–2.158)			0.973 (0.045–1.825)		
**Total Hip BMD (g/cm²)**	0.926 (0.392–1.754)			0.869 (0.320–1.608)		
	**SUA <3.9 mg/dl n(%)**	**SUA ≥3.9 mg/dL n(%)**	**p**	**SUA <3.9 mg/dl n(%)**	**SUA ≥3.9 mg/dL n (%)**	**p**
**DXA L1–L4**						
Normal	87 (12.1)	631 (87.9)		144 (15.9)	763 (84.1)	
Osteopenia	52 (15.4)	286 (84.6)	0.002	172 (19.8)	697 (80.2)	<0.001
Osteoporosis	22(25.9)	63(74.1)		107 (29.8)	252 (70.2)	
**DXA Total Hip**						0.110
Normal	82 (12.5)	574 (87.5)		186(18.1)	840 (81.9)	
Osteopenia	61(15.1)	344 (84.9)	0.042	739 (79.3)	739 (79.3)	
Osteoporosis	18(22.5)	62(77.5)		43 (24.2)	135 (75.8)	

* male participants aged <50 years (n=4)
