# Supplementary figures and images for: A Novel Approach to Determining Bone Loss Through Serum Uric Acid Levels: A Retrospective Multicenter Cohort Analysis
Source: J Clin Med. 2026 Apr 15;15(8):3020. doi: 10.3390/jcm15083020 (PMC13116610; doi:10.3390/jcm15083020)

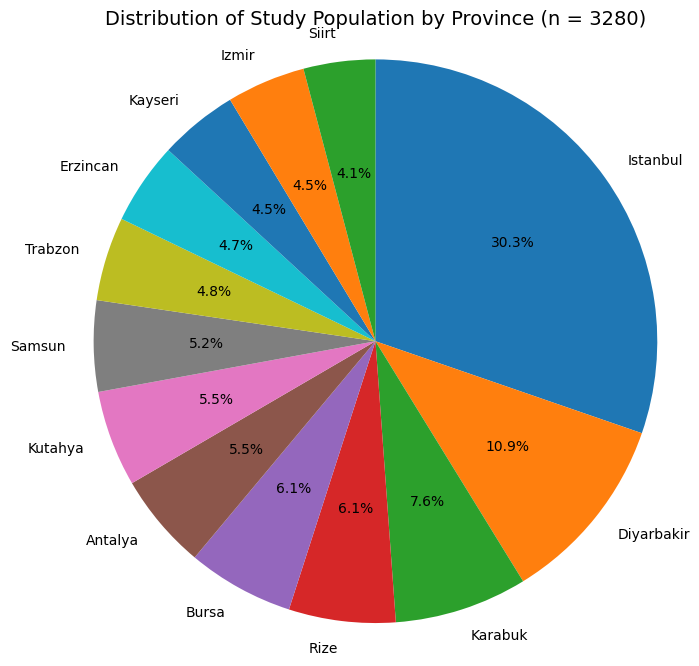

Supplement: Supplementary file 1 [file jcm-15-03020-s001.zip › Suppl. Figure S1. Study population by province.png]
